# Supplementary material for: Imaging the polymerization of multivalent nanoparticles in solution
Source: Nat Commun. 2017 Oct 2;8:761. doi: 10.1038/s41467-017-00857-1 (PMC5624893; doi:10.1038/s41467-017-00857-1)
Supplement: Supplementary file 1 — Supplementary Information [file 41467_2017_857_MOESM1_ESM.pdf]

Supplementary Figures

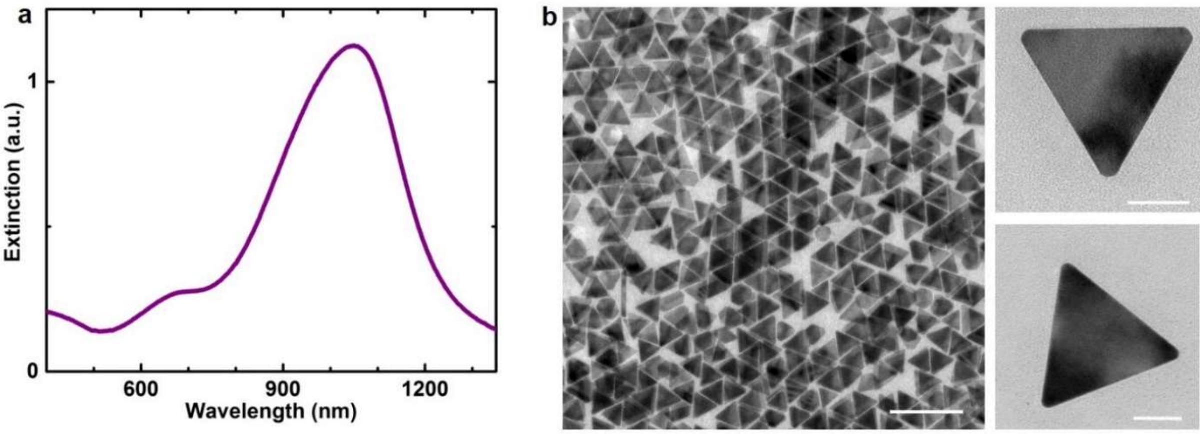

Supplementary Figure 1. Characterization of gold triangular nanoprisms. (**a**) UV-Vis-NIR spectrum of thiol-modified gold triangular nanoprisms. (**b**) The transmission electron microscopy (TEM) images of thiol-modified gold triangular nanoprisms. The TEM images in the right column show prisms with flat tips (top) and rounded tips (bottom). Scale bars: 300 nm for the low magnification image, 30 nm for the high magnification images in the right column.

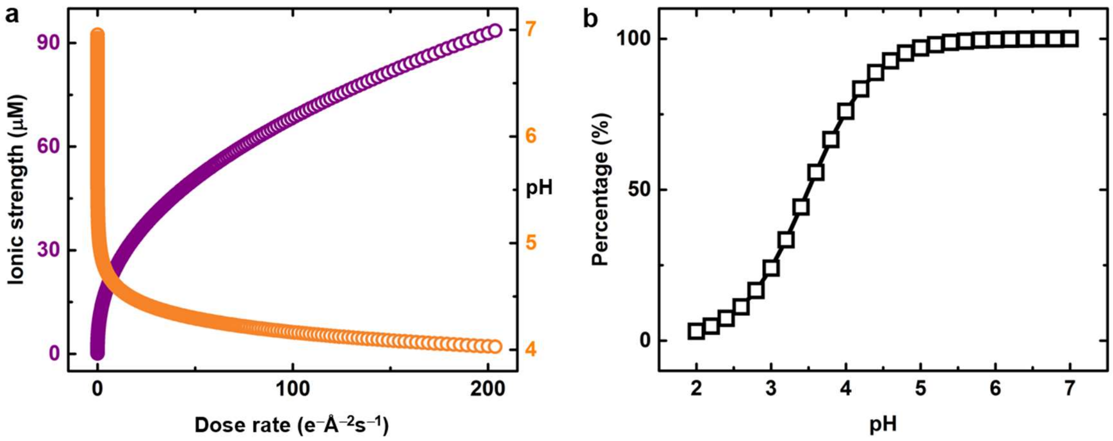

Supplementary Figure 2. Estimation of ionic strength and pH of deionized water at different dose rates, and the deprotonation percentage of thiol ligands at different pH conditions. (**a**) Changes in ionic strength (purple curve) and pH (orange curve) in deionized water at different dose rates (over the range of 0 to 200 $e^{-}Å^{-2}s^{-1}$) calculated using the computer code provided in a literature.[1] (**b**) Percentage of deprotonated, negatively charged thiol ligands at different pH conditions.

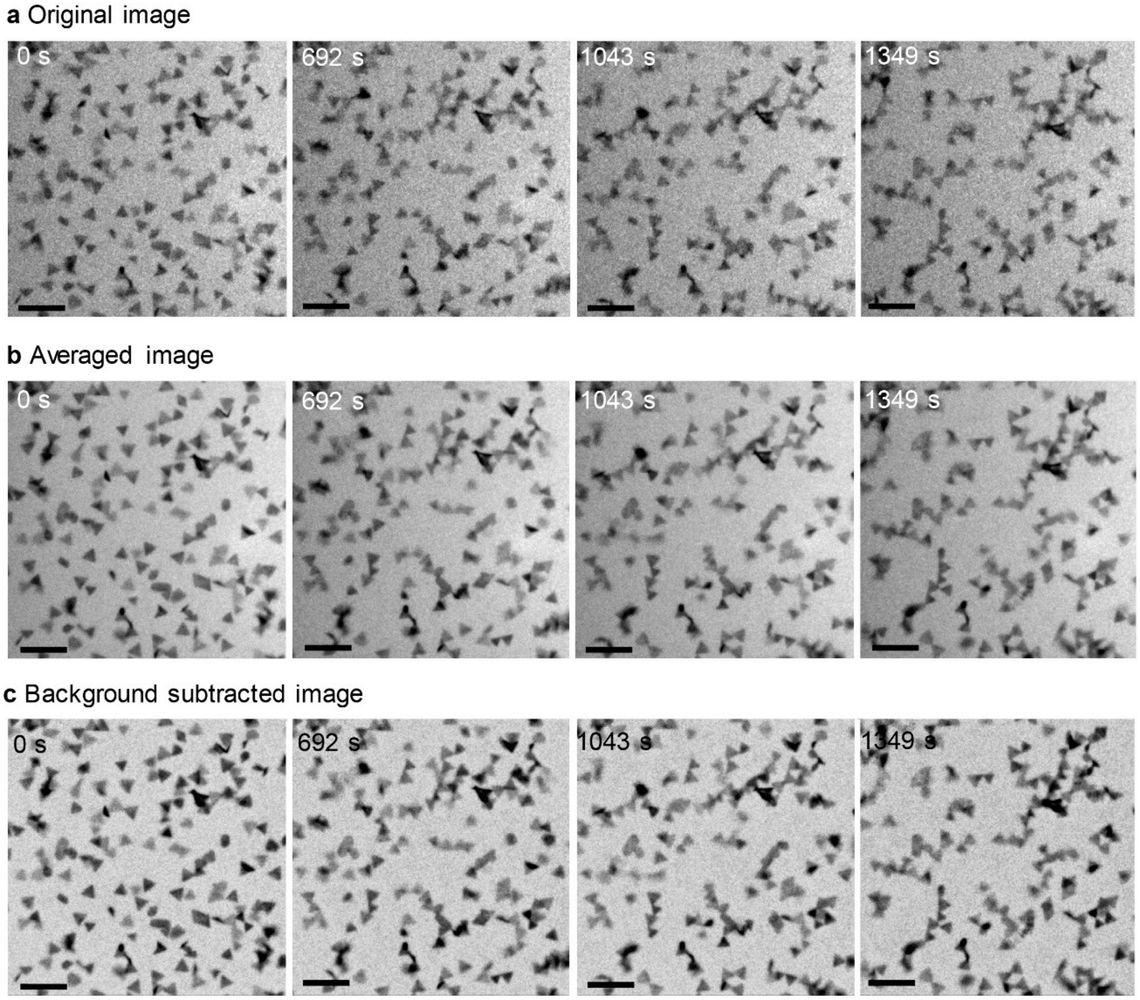

Supplementary Figure 3. Typical large-scale liquid-phase TEM snapshots for linear chain growth presented in Fig. 1c and the corresponding image processing. (**a**) Original liquid-phase TEM snapshots. (**b**) The TEM images after six neighboring sequential frames are averaged. (**c**) The TEM images after the image background is subtracted. Scale bars: 300 nm.

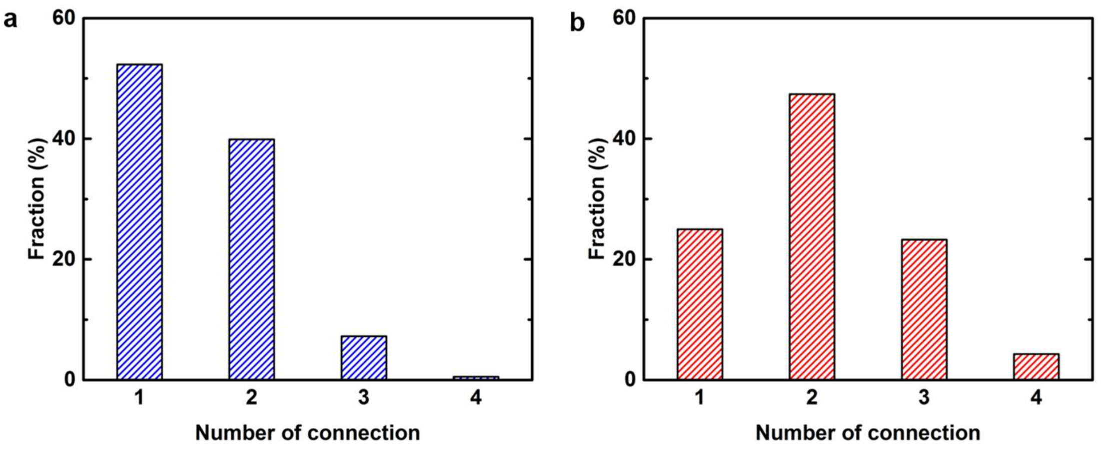

Supplementary Figure 4. The distribution of number of connections for prisms in the linear and cyclic chain assemblies. (**a**) Fraction of prisms with different numbers of connections in the linear chains (Fig. 1 and Supplementary Fig. 3). (**b**) Fraction of prisms with different numbers of connections in the cyclic chains (Fig. 5 and Supplementary Movie 4).

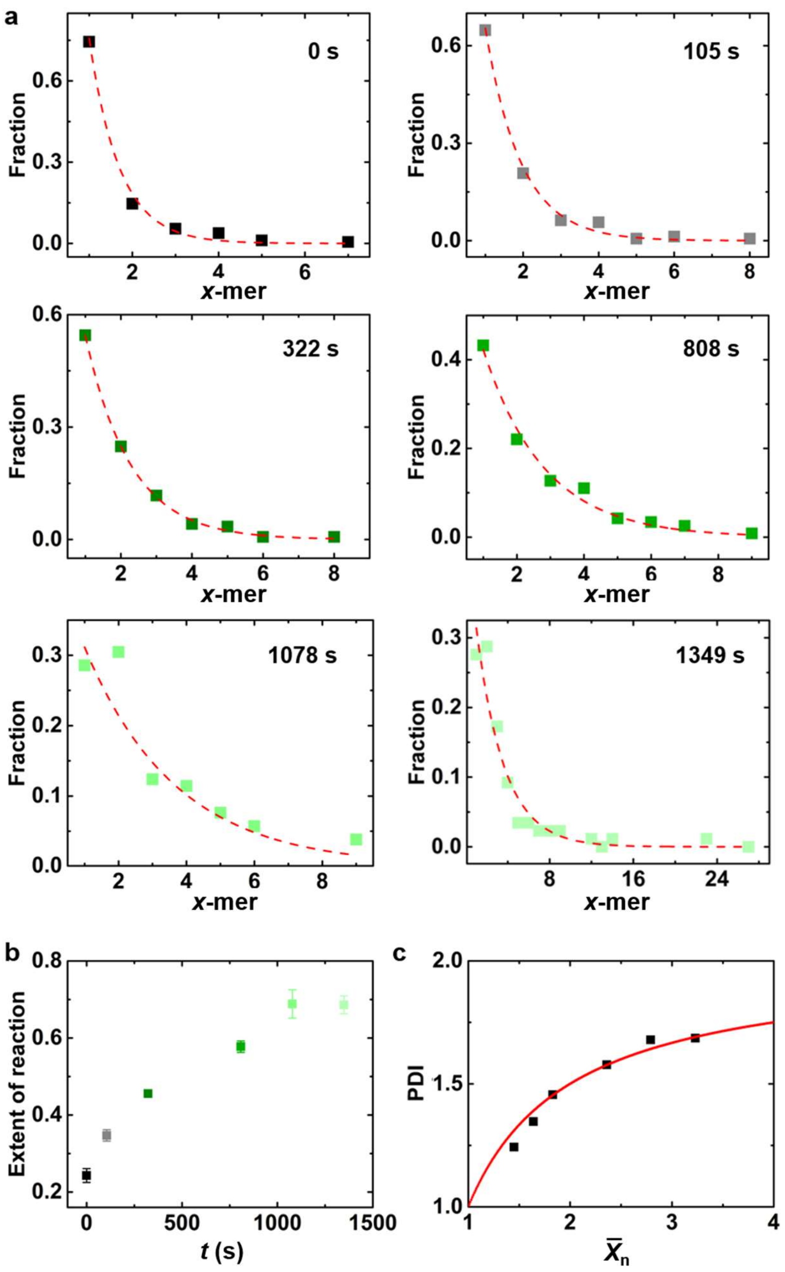

Supplementary Figure 5. Extent of reaction and polydispersity index derived from linear chain growth of prisms. (**a**) Fitted curves (red dotted line) and original data (filled squares) of the fraction of x-mers at different assembly stages for the data presented in Fig. 1 and Supplementary Fig. 3. Each color corresponds to a data point with the same color in **b**. (**b**) The graph showing the change in the extent of reaction over time t, where the extent of reaction is obtained from Flory-Schulz distribution fitting in **a**. (**c**) The graph showing polydispersity index (PDI) change as a function of $\bar{X}_\mathrm{n}$, number-average degree of polymerization, over the assembly time. The data points are shown as black squares. The red curve is the fitted line based on PDI = 2 – 1/$\bar{X}_\mathrm{n}$.

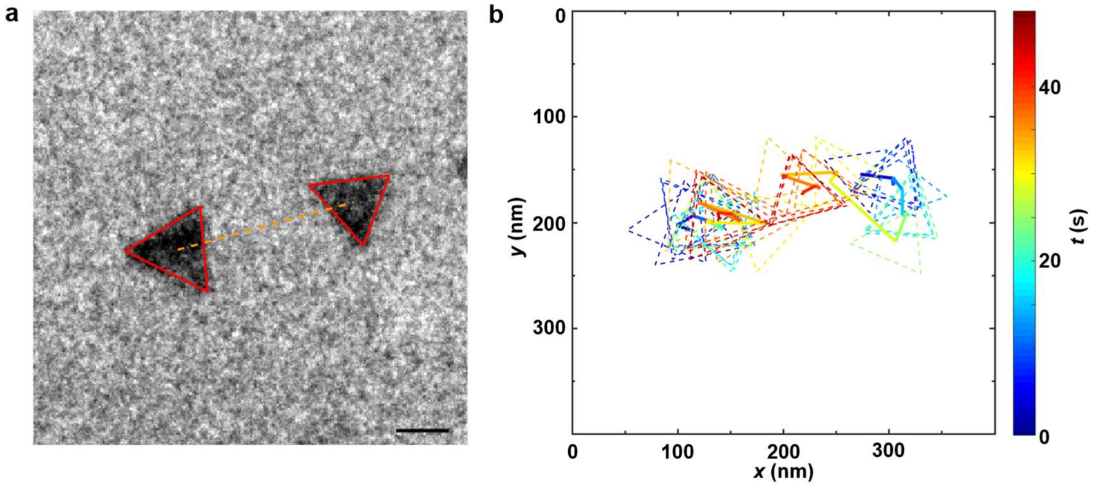

Supplementary Figure 6. Automatic image analysis of a liquid-phase TEM movie. (**a**) One TEM snapshot shows the automatically identified particle shape overlaid with an original TEM image. The red solid line shows the identified anisotropic shape, and the orange dashed line links the centers of two triangles. (**b**) Temporal evolution of two approaching prisms by overlaying automatically identified particle outlines (dashed lines) at different frames. Color of the particle outlines is coded according to time. The two solid colorful lines show the trajectories of central positions of two prisms. Scale bar: 50 nm.

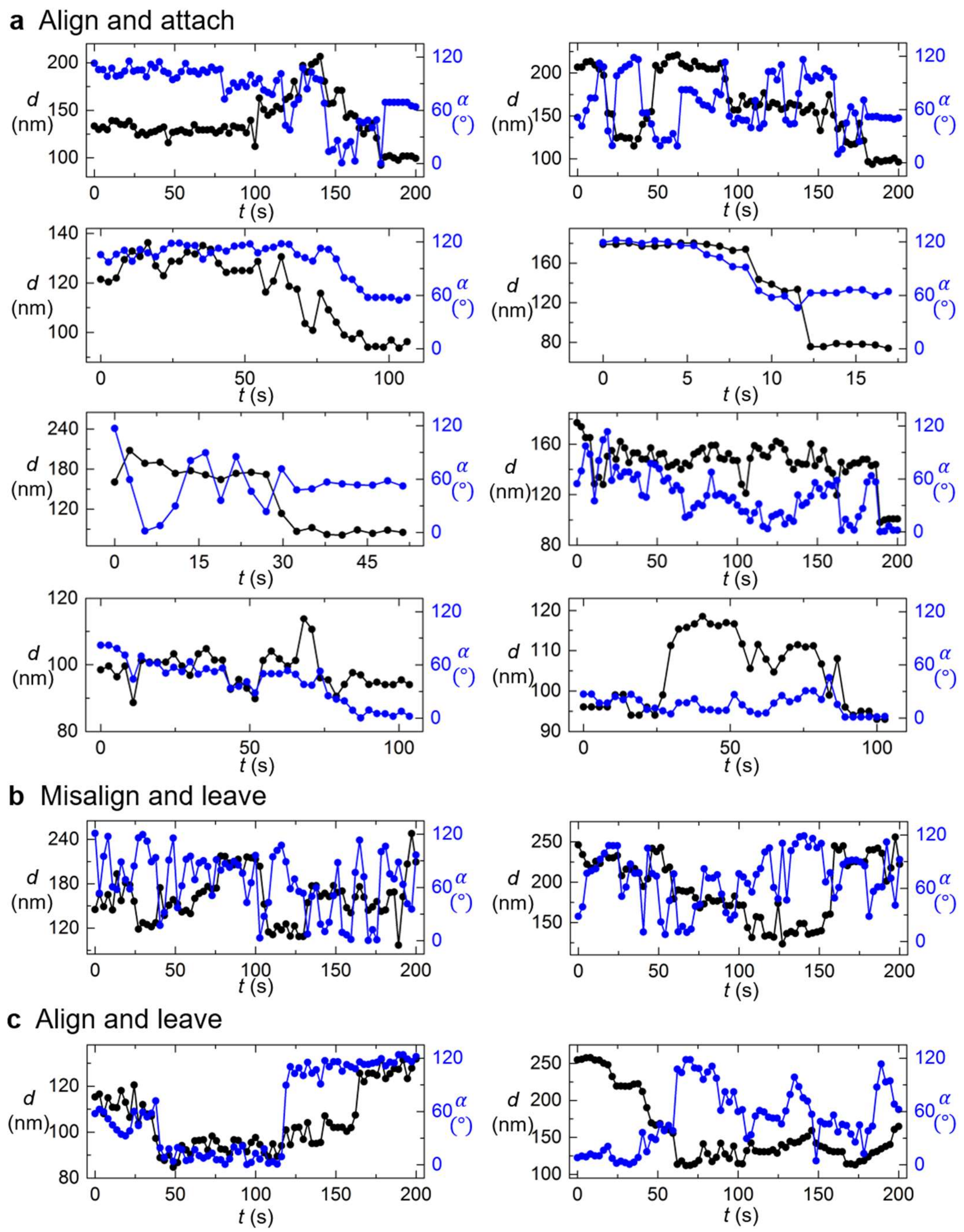

Supplementary Figure 7. Temporal traces of two approaching prisms tracked from liquid-phase TEM movies of tip-to-tip chain assembly. Graphs showing the black trace as center-to-center distance (d) between two prisms and the blue trace as relative orientation (α) for "Align and attach" (**a**), "Misalign and leave" (**b**) and "Align and leave" (**c**). Each graph records one temporal trace of a different pair of approaching prisms.

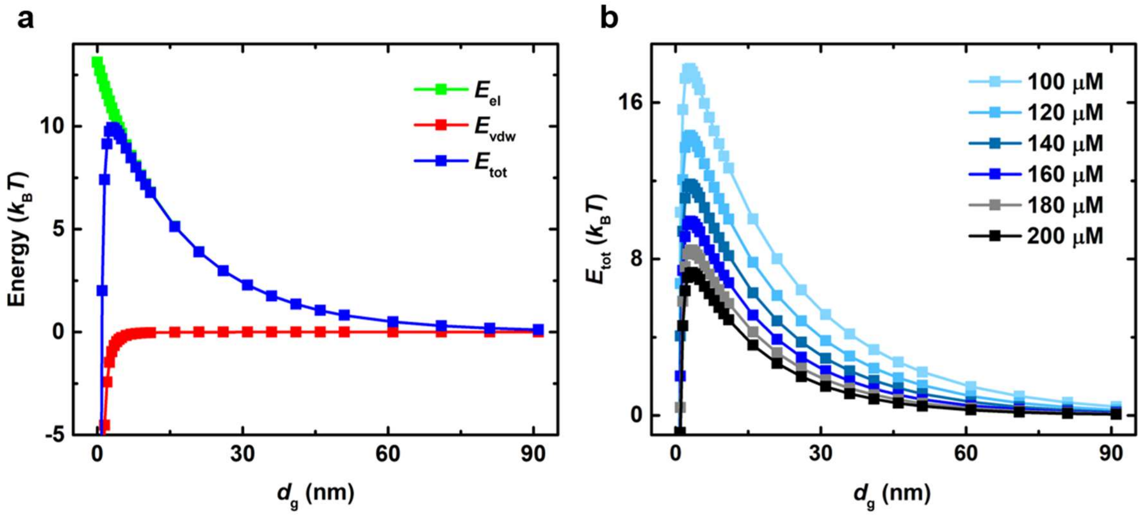

Supplementary Figure 8. Theoretical calculations of interaction energy between two prisms approaching tip-to-tip at a relative angle α = 0° at different gap distances and ionic strengths. (**a**) Electrostatic repulsion (E_{el}, green), van der Waals attraction (E_{vdw}, red) and total interaction (E_{tot}, blue) energy at different gap distances (d_g) at an ionic strength of 160 μM. The pH value of the solution is estimated as 4.51 due to radiolysis. (**b**) Dependence of total interaction energy (E_{tot}) on gap distance (d_g) at different ionic strengths at pH = 4.51.

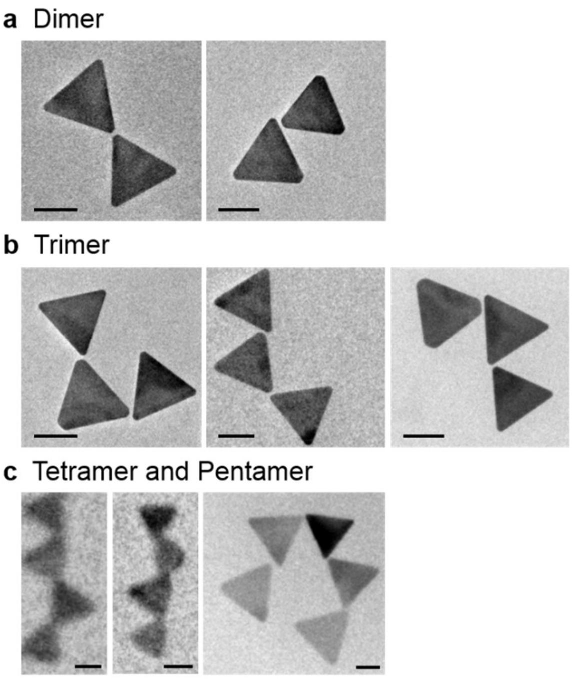

Supplementary Figure 9. TEM images of prism chains in different lengths composed of two angled bonding motifs. As shown in the TEM images, the prisms are connected either through α = 0° or α = 60° bond angles. Scale bars: 50 nm.

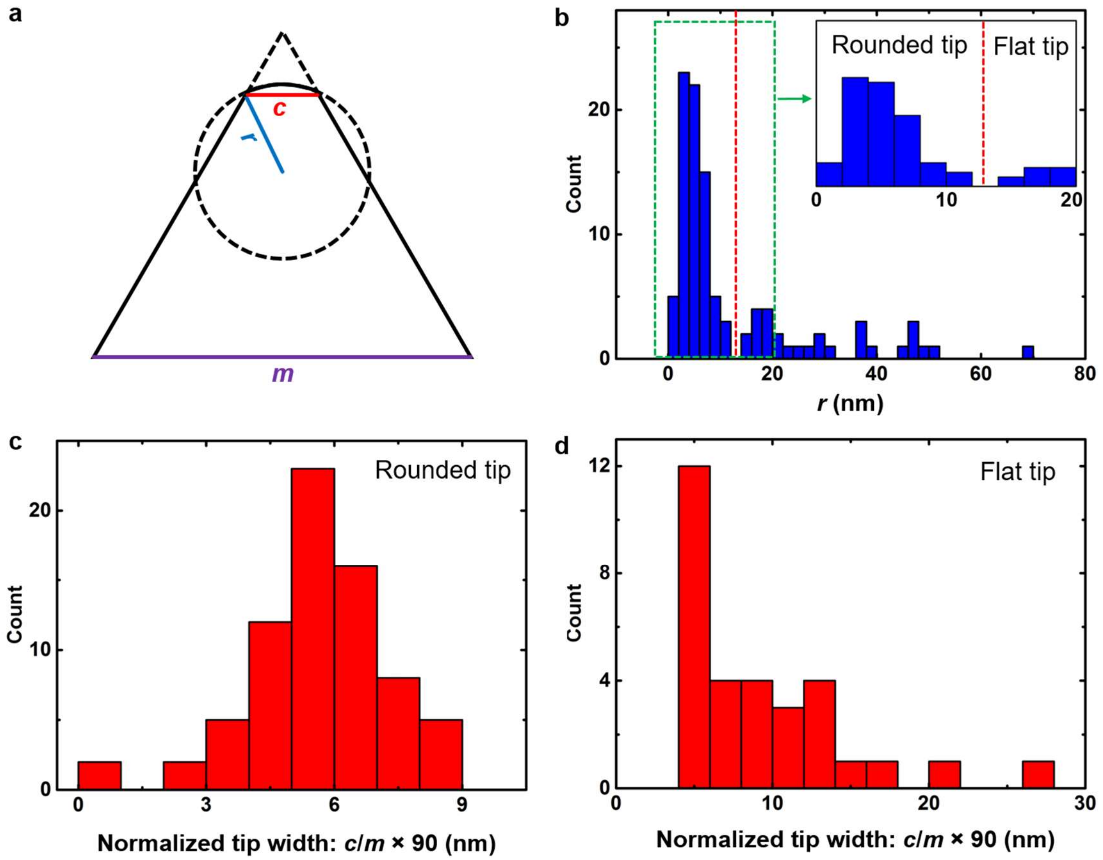

Supplementary Figure 10. Experimentally measured prism tip morphology distribution for local curvature analysis. (**a**) A schematic of the prism geometry. The radius of a circle (dotted) fitting with the tip shape is defined as r. The length of a straight line connecting two points where the fitted circle intersects with the tip area is defined as the tip width, c. The side length of the prism is defined as m. (**b**) Distribution of fitted circle radius (r) measured from 100 tips. The inset shows the distribution of the fitted circle radius (r: 0 – 20 nm, shown in the green dotted box). The rounded tip has r values smaller than ~13 nm (left to the red dotted line). The averaged r value is 5.0 ± 2.4 nm for the rounded tip, and we see tips with r values > 13 nm as flat ones. (**c**) Distribution of normalized tip width ((c / m) × 90 nm) for the rounded tip (averaged value: 5.6 ± 1.7 nm). (**d**) Distribution of normalized tip width ((c / m) × 90 nm) for the flat tip. We use the averaged value 9.3 nm in the interaction calculations.

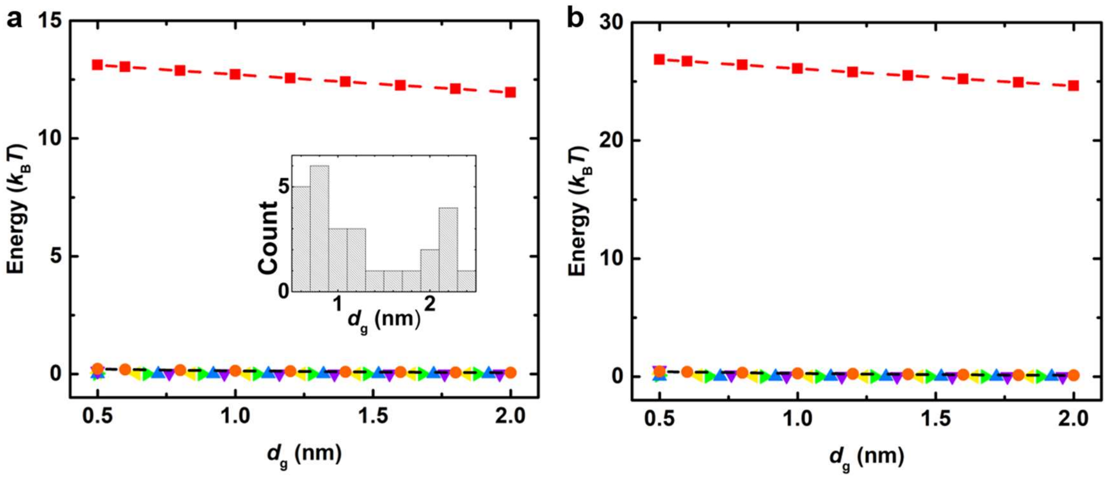

Supplementary Figure 11. Comparison between hydration and electrostatic interaction energy at different gap distances for the desired configuration of two assembled prisms. (**a**) The interaction energy curve for two prisms with rounded tips at their favored relative orientation $\alpha = 0°$ at different gap distances (d_g). (**b**) The interaction energy curve for two prisms (one round-tipped and the other flat-tipped) at their favored relative orientation $\alpha = 60°$ at different gap distances (d_g). For both (**a**) and (**b**), the red squares in the top curve of the graphs represent the electrostatic repulsion energy (the dotted red lines are the guide to the eye). The other symbols in the bottom of the graphs show the hydration interaction energy calculated based on different literature values: bare gold surfaces (orange circle);[2] thiolated gold surfaces with 3 mM *n*-decyl-β-D-glucopyranoside (yellow left triangle) or with *n*-decyl-β-D-maltopyranoside (green right triangle) in solution;[3] thiolated gold surfaces with 10^{-4} M NaCl and 10^{-5} M hexaoxyethylene dodecyl ether in solution at driving speed of 36 $nm \cdot s^{-1}$ (blue up triangle) and 55 $nm \cdot s^{-1}$ (purple down triangle).[4] The dashed black line is a guide to the eye. Detailed parameters are listed in Supplementary Table 4. Inset in (**a**): the gap distance (d_g) distribution measured from tip-to-tip assembled prisms in TEM.

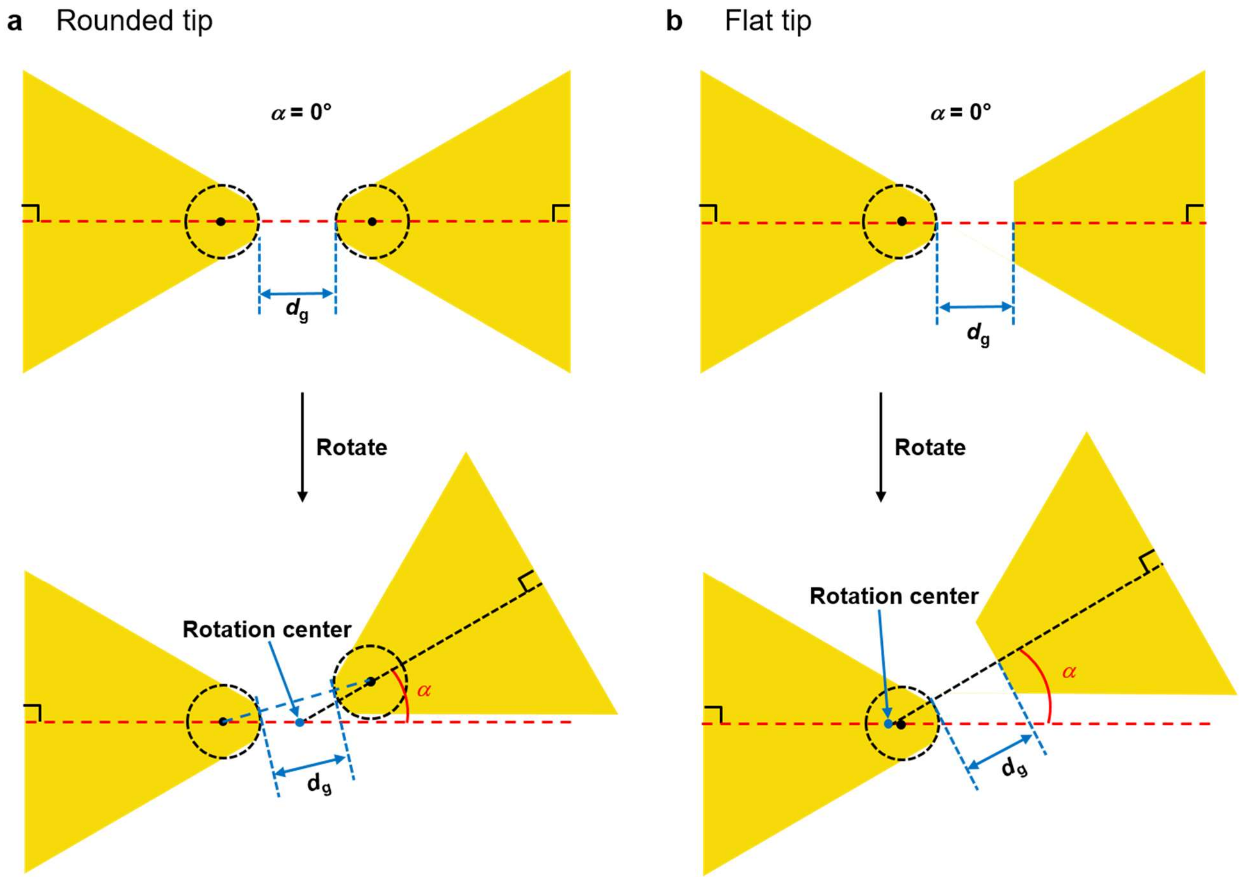

Supplementary Figure 12. Schematics of rotation centers and prism configurations for the interaction energy calculations. These schematics are most relevant for analysis in Fig. 4d and Supplementary Figs. 13-14**.** (**a**) Configurations between two round-tipped prisms at different bond angles. (**b**) Configurations between a round-tipped prism and a flat-tipped prism at different bond angles.

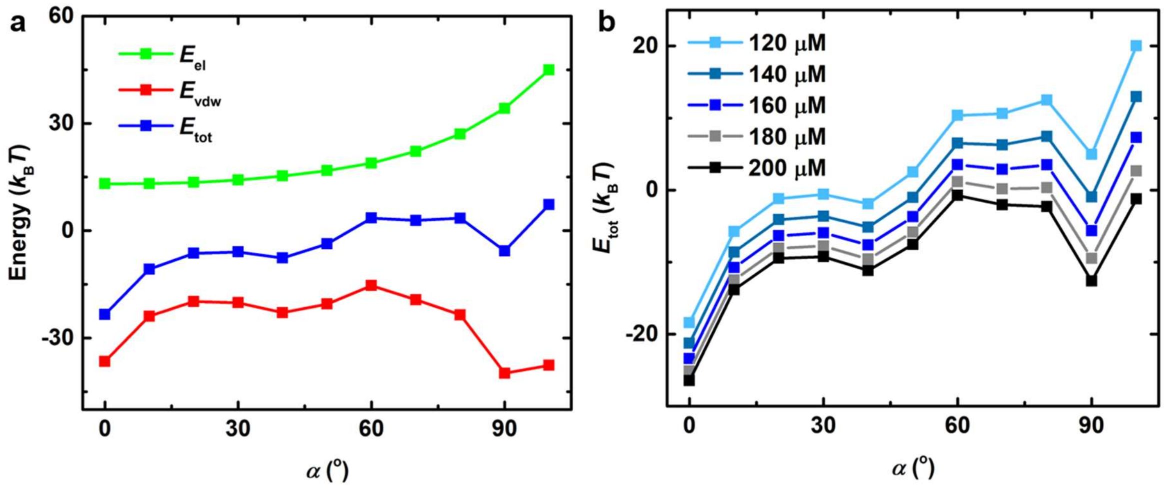

Supplementary Figure 13. Theoretical calculations of interaction energy between two tip-assembled round-tipped prisms by varying bond angle α at a given gap distance d_g of 0.5 nm. (**a**) Interaction energy calculation with different α at a total ionic strength of 160 μM at pH = 4.51. The van der Waals attraction (E_{vdw}, red), electrostatic repulsion (E_{el}, green) and total interaction (E_{tot}, blue) energy are given separately. (**b**) Dependence of total interaction energy (E_{tot}) on bond angle at different ionic strengths at pH = 4.51.

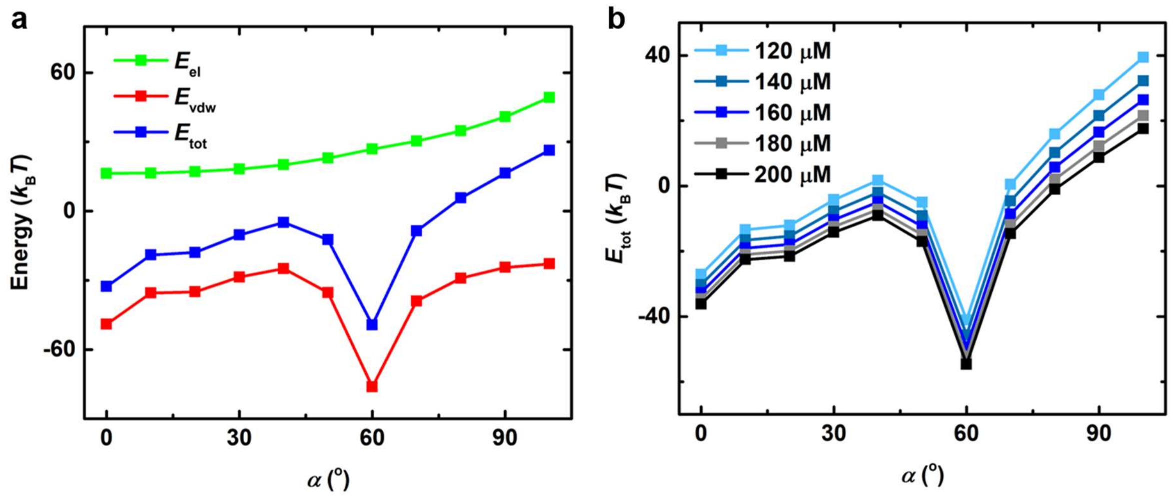

Supplementary Figure 14. Theoretical calculations of interaction energy between two tip-assembled prisms by varying bond angle α at a given gap distance d_g of 0.5 nm, one with a rounded tip and the other with a flat tip. (**a**) Energy calculation with different α at a total ionic strength of 160 μM at pH = 4.51. The van der Waals attraction (E_{vdw}, red), electrostatic repulsion (E_{el}, green) and total interaction (E_{tot}, blue) energy are given separately. (**b**) Dependence of total interaction energy (E_{tot}) on bond angle at different ionic strengths at pH = 4.51.

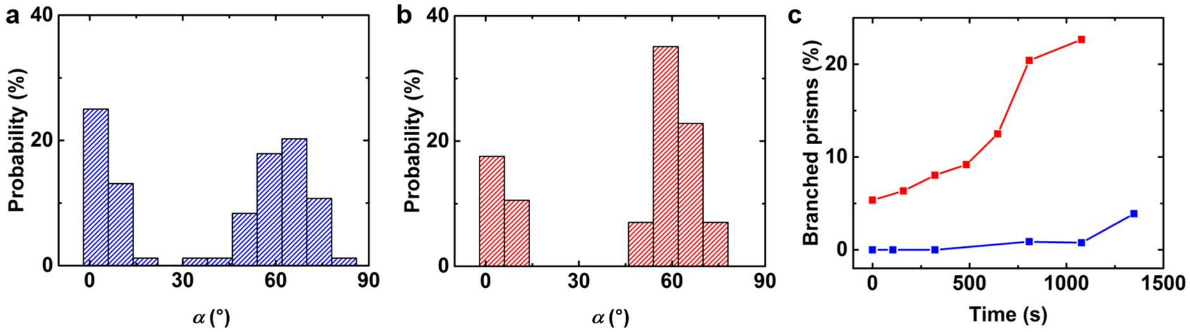

Supplementary Figure 15. Bond angle distribution and fraction of branched prisms in the linear chains and the cyclic chains. (**a**) Bond angle (α) distribution between prisms in the linear chains (Fig. 1 and Supplementary Fig. 3). (**b**) Bond angle (α) distribution between prisms in the cyclic chains (Fig. 5 and Supplementary Movie 4). (**c**) Fraction of branched prisms over the assembly time in the linear chains (blue square) and the cyclic chains (red square). The fraction is calculated by dividing the number of branched prisms (prisms whose number of connections is larger than 2) by the total number of prisms in chains.

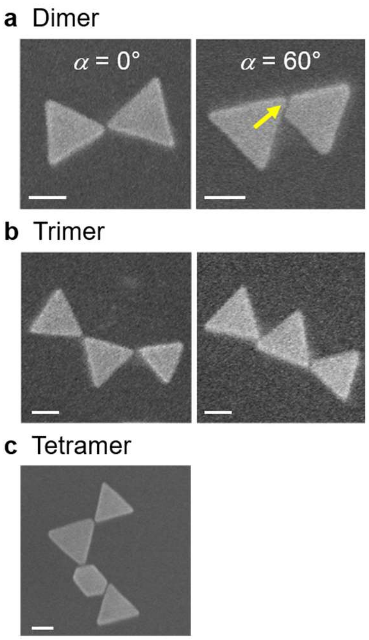

Supplementary Figure 16. Scanning electron microscopy (SEM) images of linear prism chains observed in substrate-mediated self-assembly. (**a**) The assembled dimeric prisms showing the local tip curvature determined bond angles (0° and 60°), which is consistent with the results in liquid-phase TEM experiments (Figs. 1 and 4a). (**b**) and (**c**) Prism trimers and tetramers connected tip-to-tip. Scale bars: 50 nm.

Supplementary Tables

Supplementary Table 1. Number-average degree of polymerization ($\overline{X}_n$). The $\overline{X}_n$ values measured from experimental data shown in Fig. 1e is compared with those derived from the extent of polymerization reaction in Supplementary Fig. 5b. The R-squared values in the last column are from the fitted curves in Supplementary Fig. 5b.

Time (s)	$\overline{X}_n$ (Fig. 1e)	$\overline{X}_n$ (Supplementary Fig. 5b)	Fitting R-squared
0	1.4	1.3	0.994
105	1.6	1.5	0.995
322	1.8	1.8	0.999
808	2.4	2.4	0.987
1078	2.8	3.2	0.856
1349	3.2	3.2	0.929

Supplementary Table 2. Parameters used for interaction energy calculations of linear chains (Supplementary Fig. 3) and cyclic chains (Supplementary Movie 4). The total ionic strength and pH are stabilized upon seconds after beam illumination.

Description	Supplementary Fig. 3	Supplementary Movie 4
Total ionic strength (μM)	160	205
pH	4.51	4.34
Fraction of ligand deprotonated	91%	87%
Effective charge density ($C \cdot m^{-2}$)	–0.0043	–0.0041

Supplementary Table 3. Parameters used for interaction energy calculations.

Parameter	Description	Value
σ	Diameter of gold atoms	0.332 nm
m	Prism side length	90 nm
t_{p}	Prism thickness	7.5 nm
r_{round}	Radius of the fitted circle on a rounded tip	5.0 nm
r_{flat}	Radius of the fitted circle on a flat tip	38 nm
c_{round}	Tip width of a rounded tip (obtained from the averaged normalized tip width given in Supplementary Fig. 10)	5.6 nm
c_{flat}	Tip width of a flat tip (obtained from the averaged normalized tip width given in Supplementary Fig. 10)	9.3 nm
H	Hamaker constant (gold-gold across water)	10^{-19} J
T	Temperature	298 K

Supplementary Table 4. Estimated hydration energy per unit area at contact, W_0, and decay length, δ, from literatures.[2-4] The W_0 and δ are fitted using the exponential decay function $W(D) = W_0 e^{-D/\delta}$ at the range of D of 0 – 1 nm.[5,6] Here W is the measured hydration interaction per area, W_0 is the hydration repulsion energy per area when in contact (D = 0), δ is the decay length and D is the distance between two surfaces.

W_0 ($mJ \cdot m^{-2}$)	δ (nm)	Experimental system description
0.04	1.1	Bare gold surfaces (1 mM total ionic strength at pH = 7)[2]
0.31	0.16	Thiolated gold surfaces (3 mM *n*-decyl-β-D-glucopyranoside in solution, driving speed: 22 $nm \cdot s^{-1}$)[3]
0.28	0.13	Thiolated gold surfaces (3 mM *n*-decyl-β-D-maltopyranoside in solution, driving speed: 22 $nm \cdot s^{-1}$)[3]
0.42	0.09	Thiolated gold surfaces (10^{-4} M NaCl and 10^{-5} M hexaoxyethylene dodecyl ether in solution, driving speed: 36 $nm \cdot s^{-1}$)[4]
0.34	0.21	Thiolated gold surfaces (10^{-4} M NaCl and 10^{-5} M hexaoxyethylene dodecyl ether in solution, driving speed: 55 $nm \cdot s^{-1}$)[4]

Supplementary Notes

Supplementary Note 1. Ionic strength and pH at different imaging dose rates

Under electron beam, radiolysis of pure water gives rise to radiolysis products including charged species (e.g., e^-, H^+, OH^-, HO_2^-, O^-, O_2^-, and O_3^-), which increase ionic strength and decrease pH. The degree of radiolysis is dependent on the dose rate. A rough estimation of the dependence of ionic strength and pH on dose rates was shown in Supplementary Fig. 2 following a literature,[1] assuming pre-existing ions, if any, are not involved in radiolysis reactions. The unit conversion from $e^-Å^{-2}s^{-1}$ to $Gy \cdot s^{-1}$ for the above calculations was done by multiplying the dose rate in $e^-Å^{-2}s^{-1}$ with the density normalized stopping power of water, 2.798×10^5 $eV \cdot m^2 \cdot kg^{-1}$ per electron at 200 kV.[7] Specifically, the stable ionic strengths of 160 μM for linear chains and 205 μM for cyclic chains were rationalized based on our theoretical calculations for total interaction energy between a pair of prisms, which would allow the two prisms to overcome a repulsion barrier and assemble into the prism chains (see more details in Supplementary Fig. 8 and Table 2).

Supplementary Note 2. Evaluation of pH effects on the surface charge density of prisms

The portion of negatively charged/neutral thiol ligands is determined by the pH of the solution, which determines the surface charge density of the prism. The ratio of the two states of the thiol ligands at different pH conditions was calculated by the Henderson-Hasselbalch equation, $\mathrm{pH} = \mathrm{p}K_\mathrm{a} + \log\left(\frac{[\mathrm{A}^-]}{[\mathrm{HA}]}\right)$, where $[A^-]$ is the molar concentration of negatively charged ligands (–COO^-), [HA] is that of neutral ligands (–COOH), and we use 3.5 as $\mathrm{p}K_\mathrm{a}$ ($\mathrm{p}K_\mathrm{a}$ = 3.5–3.7, provided by the manufacturer). The thiol ligand (> 1%) begins to be protonated below pH = 5.5. At pH = 4, the surface charge density decreases to 76% of the initial value (Supplementary Fig. 2b).

Supplementary Note 3. Time-lapse liquid-phase TEM image processing protocol

The time-lapse liquid-phase TEM images shown in Figs. 1c, 2b-d and 5b were processed via frame average, background subtraction and contrast adjustment. An example set of TEM images after the above image processing procedures are presented in Supplementary Fig. 3. Each frame was first extracted from TEM movies (in the format of .dm3) using an open source software ImageJ, and six sequential frames of interest were averaged by use of our customized MATLAB script. Background subtraction was performed with the averaged TEM image via ImageJ, followed by

manual adjustment of contrast. The pixel size for the background subtraction was chosen as 40, which is slightly larger than the dimension of a single prism.

Supplementary Note 4. Calculation of the step-growth polymerization parameters

The assembly rate constant (k) for the tip-to-tip prism assembly into linear chains (Fig. 1e and Supplementary Fig. 3) was derived using the rate equation for step-growth polymerization after the linear fitting of the data points shown in Fig. 1e.[8] Given $\overline{X}_{\mathrm{n}} = 4[M]_{\mathrm{o}}kt + 1$, the linear fitted line derived a slope of 0.00127 s^{-1}, with a fitting R-squared of 0.992. Then, the k value was calculated as $k = \frac{0.00127}{4[M]_{\mathrm{o}}}\ \mathrm{s}^{-1}$,

where $[M]_{\mathrm{o}} = \frac{N_{\mathrm{prism}}}{N_{\mathrm{A}}V_{\mathrm{chamber}}} = \frac{274\times10^{-3}}{6.022\times10^{23}\times2.47\times10^{-6}\times2.47\times10^{-6}\times250\times10^{-9}} = 2.98 \times 10^{-7}$ M. N_{prism} is the total number of prisms, N_{A} is the Avogadro constant, and V_{chamber} is the dimension acquired in experimental conditions for results presented in Supplementary Fig. 3. Thus, the k value was calculated as $1.1 \times 10^{3}\ \mathrm{M}^{-1}\cdot\mathrm{s}^{-1}$.

The extent of reaction was derived via Flory-Schulz distribution,[8,9] which is defined as $n_x/N_{\mathrm{L}} = (1-p)p^{x-1}$, where n_x is the number of x-mers containing x prisms, N_{L} is the total number of x-mers, and p is the extent of reaction at time t. The fraction (n_x/N_{L}) at each time t calculated from the data for the experiment presented in Supplementary Fig. 3 was plotted as a function of x (Supplementary Fig. 5a). The data points were fitted with the function of Flory-Schulz distribution, from which we derived the extent of reaction, p, at each time (Supplementary Fig. 5b). The number-average degree of polymerization ($\overline{X}_{\mathrm{n}}$) measured from Supplementary Fig. 3 (see Fig. 1e) was compared with those calculated from the extent of reaction (p), verifying the success of fitting via Flory-Schulz distribution (Supplementary Table 1). The PDI values were calculated and shown as a good fit with PDI $= 2 - \frac{1}{\overline{X}_{\mathrm{n}}}$ (Supplementary Fig. 5c).

Supplementary Note 5. Particle tracking and automatic analysis

Using a custom-developed MATLAB script, we captured not only the position of the prism, but also their anisotropic shape details from a liquid-phase TEM movie (Fig. 4c and Supplementary Fig. 6a). The automatic analysis of a TEM movie about 50 s long resolved temporal traces of two

interacting prisms (Supplementary Fig. 6b). A TEM movie with high resolution is desirable for automatic tracking of nanoparticle motions together with their shape details.

Supplementary Note 6. Prism tip contour curvature analysis

For the local curvatures of prism tips analyzed in Fig. 4c and Supplementary Fig. 10, they were measured from the TEM images of individual prisms using ImageJ and our customized MATLAB codes. The contour of the triangular surface of a single prism is determined by the image intensity gradient using customized MATLAB codes, and the corresponding local curvature was calculated from the inversed radius ($1/r$) of locally best-fitted circles (Fig. 4c).[10,11] The prism geometry parameters measured from TEM images is what we used in the calculation model (Supplementary Table 3, the prism side length was rounded to 90 nm for calculation convenience).

Supplementary Note 7. Estimation of hydration interactions

Hydration interactions occur when two surfaces immersed in water come into a sufficiently close distance, where structured water layers adsorbed or adjacent to the surfaces start to generate surface-surface interactions.[6] The range of hydration interactions is thus about a few water molecule size, about 0.1–1.4 nm.[5] Previous studies have utilized direct force measurements (surface force apparatus, atomic force microscopy, etc.) to measure the magnitude and mathematical form of hydration interactions for various solid surfaces.[2-4,12-14] Empirically, the hydration repulsion for two hydrophilic surfaces per unit area is written as $W(D) = W_0 \mathrm{e}^{-D/\delta}$.[5,6] Here W is the hydration repulsion energy per area at a distance D, W_0 is the hydration repulsion energy per area when the surfaces are in contact, δ is the decay length, and D is the distance between two surfaces. Both W_0 and δ parameters are dependent on the composition of surfaces, surface ligand chemistry (if any), and the concentration and valency of ions in the solutions. We obtained the literature values of W_0 and δ based on the hydration interaction measurements for two gold surfaces in water (Supplementary Table 4),[2-4] and estimated the corresponding hydration interactions following the empirical equation. We chose a gap distance ranging from 0.5 nm to 2 nm for the estimation of hydration interaction in our system, based on the gap distance values we observed (0.5–2.4 nm, the inset distribution in Supplementary Fig. 11). We found that the hydration interaction between two tip-to-tip assembled prisms are constantly below 1 $k_\mathrm{B}T$, much smaller than the electrostatic repulsion (Supplementary Fig. 11). Thus, in our interaction

calculation that explains the bimodal bond angle distribution, we considered only van der Waals attraction and electrostatic repulsion, which indeed correctly predicted our experimental observation.

Our estimation that the hydration interaction is negligible for the gold prisms coated with charged organic ligands is consistent with previous studies. Previous force measurements[2,15,16] have shown that if the solid surfaces are coated with charged ligands like in our work (other than H^+ or OH^- ions, generic for oxides), hydration interactions are negligible due to three reasons: the ligands strongly interact with surface water layers and disturb the liquid structuring; the ligands prevent the surfaces from getting close enough to fall into the short range of hydration interaction; the charged ligands bring strong electrostatic repulsion into the system which obscures hydration interaction. Likewise, previous work on metallic nanoparticle growth and coalescence[17,18] have shown that when nanoparticles are coated with charged ligands (cetyltrimethylammonium bromide),[17] hydration interactions are negligible in determining assembly configurations, but when the nanoparticles are naked (no ligand coating), hydration interactions are significant.[17,18] Note that in our literature survey, we did not find hydration interaction measurements for gold surfaces coated with the same charged ligands as the thiol ligands in our sample. We used the available literature values for either naked gold[2] or nonionic ligand coated gold surfaces,[3,4] which actually render our estimated hydration interactions (Supplementary Fig. 11) higher than the actual values for our gold surfaces coated with charged ligands, which further suggests that hydration interaction does not influence the assembly configurations in our system.

Supplementary Note 8. Analysis of branched prisms in assembled chains

The branched prisms were counted from the linear chain assemblies (Fig. 1 and Supplementary Fig. 3) and cyclic chain assemblies (Supplementary Movie 4). We defined the branched prism as a prism that is connected with more than two neighboring prisms in chains. At different assembly times, we counted the total number of prisms and branched prisms in chains, and obtained the fraction of branched prisms by dividing the number of branched prisms by the total number of prisms in chains (Supplementary Fig. 15c).

Supplementary References

1 Schneider, N. M. *et al.* Electron-water interactions and implications for liquid cell electron microscopy. *J. Phys. Chem. C* **118**, 22373-22382 (2014).

2 Giesbers, M., Kleijn, J. M. & Cohen Stuart, M. A. The electrical double layer on gold probed by electrokinetic and surface force measurements. *J. Colloid Interface Sci.* **248**, 88-95 (2002).

3 Persson, C. M. & Kumpulainen, A. J. Surface forces measured in sugar surfactant solutions between two hydrophobic thiolated silica spheres. *Colloids Surf., A* **233**, 43-49 (2004).

4 Stubenrauch, C., Rojas, O. J., Schlarmann, J. & Claesson, P. M. Interactions between nonpolar surfaces coated with the nonionic surfactant hexaoxyethylene dodecyl ether $C_{12}E_6$ and the origin of surface charges at the air/water interface. *Langmuir* **20**, 4977-4988 (2004).

5 Butt, H.-J., Cappella, B. & Kappl, M. Force measurements with the atomic force microscope: technique, interpretation and applications. *Surf. Sci. Rep.* **59**, 1-152 (2005).

6 Israelachvili, J. N. *Intermolecular and Surface Forces*. (Academic press, 2011).

7 Alloyeau, D. *et al.* Unravelling kinetic and thermodynamic effects on the growth of gold nanoplates by liquid transmission electron microscopy. *Nano Lett.* **15**, 2574-2581 (2015).

8 Liu, K. *et al.* Step-growth polymerization of inorganic nanoparticles. *Science* **329**, 197-200 (2010).

9 Flory, P. J. *Principles of Polymer Chemistry*. (Cornell University Press, 1953).

10 Pratt, V. Direct least-squares fitting of algebraic surfaces. *SIGGRAPH Comput. Graph.* **21**, 145-152 (1987).

11 Ye, X. *et al.* Single-particle mapping of nonequilibrium nanocrystal transformations. *Science* **354**, 874-877 (2016).

12 Grabbe, A. & Horn, R. G. Double-layer and hydration forces measured between silica sheets subjected to various surface treatments. *J. Colloid Interface Sci.* **157**, 375-383 (1993).

13 Parsegian, V. A. & Zemb, T. Hydration forces: observations, explanations, expectations, questions. *Curr. Opin. Colloid Interface Sci.* **16**, 618-624 (2011).

14 Ninham, B. W., Duignan, T. T. & Parsons, D. F. Approaches to hydration, old and new: insights through Hofmeister effects. *Curr. Opin. Colloid Interface Sci.* **16**, 612-617 (2011).

15 Grabbe, A. Double layer interactions between silylated silica surfaces. *Langmuir* **9**, 797-801 (1993).

16 Chapel, J.-P. History-dependent hydration forces measured between silica surfaces. *J. Colloid Interface Sci.* **162**, 517-519 (1994).

17 Anand, U., Lu, J., Loh, D., Aabdin, Z. & Mirsaidov, U. Hydration layer-mediated pairwise interaction of nanoparticles. *Nano Lett.* **16**, 786-790 (2016).

18 Welch, D. A. *et al.* Understanding the role of solvation forces on the preferential attachment of nanoparticles in liquid. *ACS Nano* **10**, 181-187 (2016).
