# Supplementary material for: Imaging the polymerization of multivalent nanoparticles in solution
Source: Nat Commun. 2017 Oct 2;8:761. doi: 10.1038/s41467-017-00857-1 (PMC5624893; doi:10.1038/s41467-017-00857-1)
Supplement: Supplementary file 3 — Description of Additional Supplementary Information [file 41467_2017_857_MOESM3_ESM.pdf]

Description of Additional Supplementary Files

File Name: Supplementary Movie 1
Description: **Two gold nanoprisms align and then assemble tip-to-tip**. This synchronized movie includes a liquid-phase TEM movie (left) of two prisms moving in the solution, the tracked shape contours of the prisms (right), and a graph of the prism-prism distance as well as their relative orientation over time (bottom). The movie shows that two prisms are pre-aligned first and then assembled in the tip-to-tip configuration. The movie is played at 3.3 frames per sec (fps), 2.5× real time. Scale bar: 100 nm.

File Name: Supplementary Movie 2
Description: **Two misaligned nanoprisms come close and then apart.** This synchronized movie includes a liquid-phase TEM movie (left) of two moving prisms, and a graph of the prism-prism distance as well as their relative orientation over time (right). The movie shows that two prisms approach each other in the misaligned configuration and are then repelled apart. The movie is played at 7.4 fps, 20× real time. Scale bar: 100 nm.

File Name: Supplementary Movie 3
Description: **Two nanoprisms align and then leave apart.** This synchronized movie includes a liquid-phase TEM movie (left) and a graph of the prism-prism distance as well as their relative orientation over time (right). The movie shows that two prisms are pre-aligned as they approach and still leave apart, indicating a repulsion effect. The movie is played at 7.4 fps, 20× real time. Scale bar: 100 nm.

File Name: Supplementary Movie 4
Description: **The self-assembly of cyclic chains from nanoprisms.** This synchronized movie includes a liquid-phase TEM movie (left) and a plot of branched prism fraction over time (right). This fraction is calculated by dividing the number of branched prisms (prisms whose number of connections is large than 2) by the total number of prisms in x-mers. The first part of the TEM movie is played at 5.92 fps, 80× real time. The second part of the movie (12 mins later) is played at 1.48 fps, 20× real time. Scale bar: 150 nm.
